# CD39 identifies a specific CD8 + T cell population in lung adenocarcinoma-related metastatic pleural effusion

**DOI:** 10.1186/s12865-023-00590-z

**Published:** 2023-12-12

**Authors:** Lei-lei Lv, Hong-bin Wang, Yao-xin Zhang, Jia-wei Zhai, Yu Shen, Qiu-Xia Qu, Cheng Chen

**Affiliations:** 1 Department of Respiratory and Critical Medicine, The First Affiliated Hospital of Soochow University, 899 Pinghai Road, Suzhou, 215006 China; 2 Clinical Immunology Institute, The First Affiliated Hospital of Soochow University, 178 Ganjiang Road, Suzhou, 215006 China

**Keywords:** CD39, CD8, Malignant pleural effusion, EGFR, Lung cancer

## Abstract

Malignant pleural effusion (MPE), which is a complex microenvironment that contains numerous immune and tumour signals, is common in lung cancer. Gene alterations, such as driver gene mutations, are believed to affect the components of tumour immunity in the microenvironment (TIME) of non-small-cell lung cancer. In this study, we have shown that pleural CD39 + CD8 + T cells are selectively elevated in lung adenocarcinoma (LUAD) with wild-type epidermal growth factor receptor (EGFR^wt^) compared to those with newly diagnosed mutant EGFR (EGFR^mu^). Furthermore, these CD39 + CD8 + T cells are more prevalent in MPE with acquired resistance to EGFR-tyrosine kinase inhibitors (AR-EGFR-TKIs). Our analysis reveals that pleural CD39 + CD8 + T cells exhibit an exhausted phenotype while still retaining cytolytic function. Additionally, they have a higher T cell receptor (TCR) repertoire clonality compared to CD39-CD8 + T cells, which is a unique characteristic of LUAD-related MPE. Further investigation has shown that TCR-Vβ clonality tends to be more enhanced in pleural CD39 + CD8 + T cells from MPE with AR-EGFR-TKIs. In summary, we have identified a subset of CD8 + T cells expressing CD39 in MPE, which may potentially be tumour-reactive CD8 + T cells. This study provides new insights into the dynamic immune composition of the EGFR^mu^ tumour microenvironment.

## Introduction

Malignant pleural effusion (MPE) is a common complication of advanced lung cancer, caused by tumour metastasis to the pleural space [1]. In MPE, an accumulation of lymphocytes often occurs, which may represent an alternative microenvironment of the tumour microenvironment (TME) [2]. However, it is important to determine whether a cancer therapy will have the same immune-modulating effects in both MPE and the primary sites. Additionally, the role of suppressive immune cells related to tumour-driver genes in non-small-cell lung cancer (NSCLC) patients has not been fully established [3]. A better understanding of the pathogenesis of MPE could lead to the development of more effective therapeutic options.

More than 60% of NSCLCs express epidermal growth factor receptor (EGFR), making it an important therapeutic target for these tumours. EGFR-mutant (EGFR^mu^) lung adenocarcinoma (LUAD) has a distinct TME compared to EGFR wild-type (EGFR^wt^) LUAD. EGFR^mu^ tumour cells can recruit various immunosuppressive cells by secreting cytokines. The lack of proinflammatory cells, the presence of suppressive cell types, and the lower expression of immune checkpoint proteins may contribute to the immune-silent environment of EGFR^mu^ LUAD. Additionally, EGFR-TKI treatment was associated with changes in the TME of EGFR^mu^ LUAD. The expression of programmed cell death-ligand 1 (PD-L1) in tumour cells was significantly increased and tumour mutation burden tended to be increased after the development of resistance to EGFR-TKI treatment, which simultaneous affects the T cell receptor (TCR) clonality of tumour-infiltrating lymphocytes (TILs).

CD39 is an ectonucleotidase expressed by B cells, innate cells, regulatory T cells, as well as activated CD4 + and CD8 + T cells, which can result in local production of adenosine, leading to an immunosuppressive environment [4–7]. Recent evidence suggests the expression of CD39 on CD8 + T cells as a marker of exhausted TILs [4, 8]. A recent study demonstrated the co-expression of CD39 with the marker of resident memory CD8 + T cells, suggesting a protective role for these cells in cancer survival [9, 10]. Moreover, CD39 has also been reported as a useful marker to discriminate antigen-specific from nonspecific bystander CD8 + T cells in a tumour environment, providing an approach to identify tumour-reactive CD8 + T cells, with important implications for developing future therapeutic strategies [11–13]. However, the functional characteristics by which CD39 + CD8 + T cells infiltrate lung cancer-related MPE remain unknown.

Our previous studies have demonstrated an increased frequency of CD8 + T cells in MPE compared to peripheral blood among lung cancer patients. These patients contained more of the effector memory subset (Tem) and central memory subset (Tcm). Additionally, MPE-derived Tem and Tcm subsets expressed more CD39, along with higher cytokine production [14]. In this present study, we further investigated the accumulation of CD39 + CD8 + T cells in MPE and explored the possibility of co-occurring genetic alterations as its determinant, especially EGFR. This research aims to characterise MPE-derived CD39 + CD8 + T cells as a potential target for immunotherapy and understand the relationship between targeted therapy and immunotherapy in NSCLC.

## Materials and methods

### Patients

A total of 125 subjects were included in this study and were classified according to various criteria, as presented in Tables 1 and 2. Routine laboratory diagnostic procedures were performed on these patients, and their samples were subjected to conventional cytological analysis. Effusions were classified as malignant if the cytological examination confirmed the presence of malignant cells. Diagnosis of tuberculosis was based on either positive culture findings of Mycobacterium tuberculosis (in pleural fluid or other biological material) or the presence of granulomatous lesions in pleural biopsy specimens. Exudative pleural effusion was diagnosed based on criteria such as protein levels, LDH levels, and pleural fluid cell counts, which were all from patients with cardiac insufficiency.

**Table 1:** Subject characteristics

Variable	Number (*n* = 125, %)
**Sex**	
Male	84 (67.2)
Female	41 (32.8)
**Age (years)**	
< 65	45 (36)
≥ 65	80 (64)
**Pathology**	
**Tuberculosis**	**11 (8.8)**
**Exudative**	**16 (12.8)**
**Malignant**	**98 (78.4)**
NSCLC	70 (56)
Adenocarcinoma	63 (50.4)
Squamous	7 (5.6)
SCLC	8 (6.4)
Others	14 (11.2)

**Table 2:** Characteristics of patients with EGFR mutation

EGFR mutation status	Number (*n* = 31, %)		
	**Total**	**Firstly-diagnosed**	**Recurrent**
exon 19 deletion	18(58.06)	7(22.58)	11(35.48)
exon 21 L858R mutation	13(41.94)	5(16.13)	8(25.81)

Subjects with autoimmune diseases (e.g., rheumatoid arthritis, systemic lupus erythematosus), chronic infections (e.g., human immunodeficiency virus infection), and individuals who had received immunotherapy were excluded from the study. The research protocol was approved by the ethics committee of the First Affiliated Hospital of Soochow University.

### Pleural effusion preparation

Fresh pleural effusion samples were immediately transported to the laboratory under 4 °C conditions for testing. Mononuclear cells were isolated from fresh pleural effusions using histopaque gradient centrifugation. In brief, the fluid was incubated with 1X RBC lysis buffer, which was diluted from 10X RBC lysis buffer (BioLegend, USA) to remove red blood cells, and then centrifuged in 50 ml tubes at 1800 rpm for 5 min. After two washes with phosphate-buffered saline (PBS), the mononuclear cell band was carefully transferred into a conical centrifuge tube, with PBS added for later use.

### Flow cytometry (FCM) analysis

Mononuclear cell suspensions were prepared at a concentration of 2 × 10^6^ cells/ml for immune staining. A total of 100 μL aliquots were transferred to polypropylene test tubes, and conjugated monoclonal antibodies (mAb) or isotype controls were added to each tube. Multiparametric FCM was performed to determine cell marker expression. The following fluorochrome-conjugated mAb were used: CD45 (APC-conjugated anti-CD45, BioLegend), CD8 (APC-Cyanine7-conjugated anti-CD8, BioLegend), CD39 (PE-Cyanine7-conjugated anti-CD39, BioLegend), PD-1 (APC-conjugated anti-PD-1, BioLegend), and Tim-3 (PE-conjugated anti-Tim-3, BioLegend).

### Intracellular staining

For ex vivo intracellular IFN-γ and TNF-α detection, freshly isolated mononuclear cell suspensions were cultured in a complete RPMI 1640 medium containing PMA (50 ng/ml) and ionomycin (500 ng/ml) for 3 h. Cells were then fixed and permeabilised (eBioscience™ Foxp3/Transcription Factor Staining Buffer Set, ThermoFisher) for 30 min and 40 min, respectively, and washed with a permeabilisation buffer (ThermoFisher). Next, cells were stained with FITC-conjugated anti-IFN-γ mAb (eBioscience) and PE-conjugated anti-TNF-α mAb (BioLegend) for 30 min at 4 °C. Multi-coloured FCM analyses were performed on Cytoflex (Beckman). Data were analysed with FlowJo software (Tree Star).

### Analysis of the TCR-Vβ repertoire

We evaluated the usage of the TCR-Vβ family within CD39 + CD8 + T cell subsets through flow cytometric analysis using the IOTest Beta Mark TCR Repertoire Kit® (Beckman Coulter, Marseille, France). This kit comprises monoclonal antibodies designed to identify 24 distinct TCR-Vβ families. Each set consists of three different reserved anti-Vβ family-specific mAb labelled with FITC, PE, or both. Furthermore, we calculated the means and standard deviations (SD) of TCR-Vβ family usage to compare the differences between CD39 + and CD39-CD8 + T cells. The specific value of TCR-Vβ usages is then translated into the number of SD. Heat maps of TCR-Vβ usages of CD39 + CD8 + T cells and CD39-CD8 + T cells were created based on SD. In these heat maps, squares transitioning from green to red represent values exceeding the lower (–1 SD) to upper (+ 6 SD) limits of the TCR-Vβ family, respectively.

### Statistics

Statistical analysis was performed using GraphPad Prism 5 (GraphPad, La Jolla, CA). Paired t-tests were used for matched samples, and two-sided t-tests were applied for continuous variables. Receiver operating characteristic (ROC) curves were calculated to determine the cut-off level for the ratio of pleural CD39 + CD8 + T cells, indicating the occurrence of EGFR mutations in adenocarcinoma. All tests were two-sided, and a *P*-value of less than 0.05 was considered statistically significant.

## Results

### Baseline characteristics of patients

A total of 125 eligible patients were enrolled, and their baseline characteristics are presented in Table 1. The patient cohort consisted of 91 males and 34 females, with a median age of 67 years (range: 17–90 years). Among the patients, 63 had LUAD, 13 had small cell lung cancer, 8 had lung squamous cancer, 14 had other malignant diseases (including 3 with mesothelioma, 5 with adenosquamous carcinoma, and 6 with metastatic carcinoma), 11 had tuberculosis, and 16 had exudative pleural effusion.

### Accumulation of CD39 + CD8 + T cells in MPE.

**Fig. 1:**
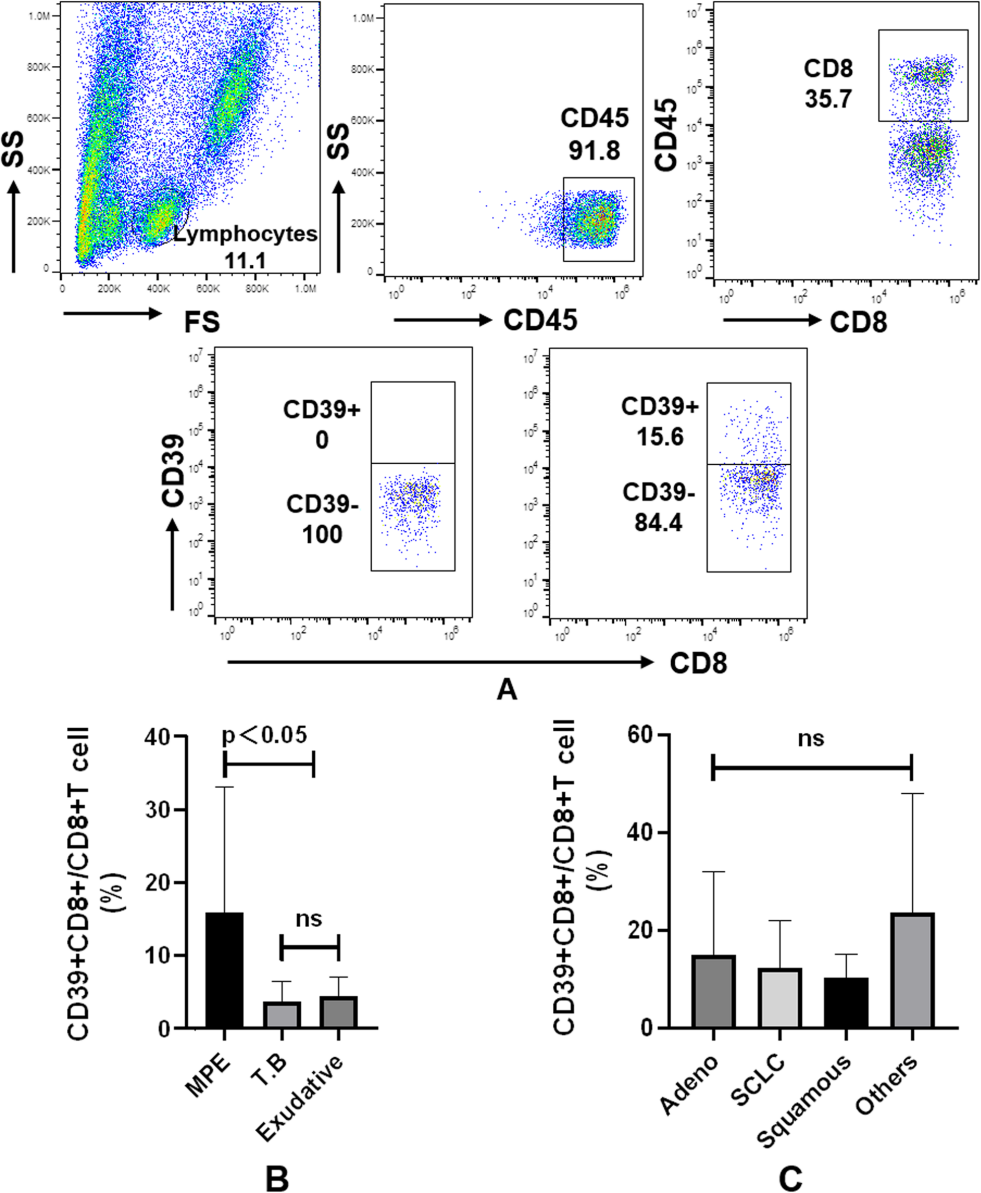
Analysis of CD39 + CD8 + T cells derived from pleural effusion by FCM. **A** Representative dot plots are shown. **B** CD39 + CD8 + T cells were significantly more abundant in MPE (15.97 ± 1.72%) compared to tuberculosis (3.74 ± 0.80%, *P* = 0.03) and exudative pleural effusion (4.47 ± 0.66%, *P* = 0.02). C) CD39 + CD8 + T cells showed comparable frequencies among different pathological types of MPE

First, we employed multiparametric FCM to identify CD39 + CD8 + T cells in all types of pleural effusions (Fig. 1A). By calculating the levels of CD39 expression as CD39^pos^ and CD39^neg^, we observed that the percentages of CD39 + CD8 + T cells were significantly higher in MPE (15.97 ± 1.72%) compared to those in tuberculosis (3.74 ± 0.80%, *P* = 0.03) and exudative pleural effusion (4.47 ± 0.66%, *P* = 0.02) (Fig. 1B). Additionally, we analysed this cell population in MPE across different pathological types (Fig. 1C). Our findings indicated that CD39 + CD8 + T cells were detected at similar frequencies in adenocarcinoma-related MPE (15.29 ± 2.14%) when compared to small cell lung cancer (12.40 ± 2.66%, *P* = 0.94) and squamous cancer (10.30 ± 1.73%, *P* = 0.86). These data demonstrate the accumulation of double-positive CD39 + CD8 + T cells in MPE, which may play a role in the regional tumour immune response.

### Impact of EGFR status on the accumulation of CD39 + CD8 + T cells in LUAD-related MPE

Given the recognised influence of EGFR gene mutations on the components of the tumour immunity in the microenvironment in NSCLC, we investigated whether the accumulation of pleural CD39 + CD8 + T cells was associated with the EGFR status in LUAD-related MPE. We obtained data about EGFR status from medical records (2019–070). Among those LUAD patients(*n* = 63), 59 patients were definitively identified with EGFR gene status (Table 2). They were further categorised into those with EGFR^wt^ (*n* = 28) and those with mutations (exon 19 deletion/exon 21 L858R mutation, *n* = 31). Among the patients with EGFR^mu^, it was subdivided into initially-diagonalized (*n* = 12) and recurrent cancer (*n* = 19).

**Fig. 2:**
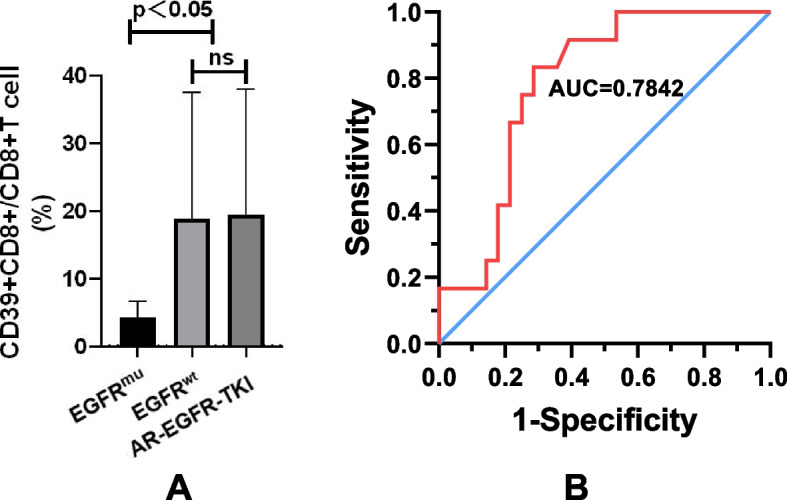
EGFR mutation and infiltration of CD39 + CD8 + T cell population in MPE of LUAD. **A** The accumulation of CD39 + CD8 + T cells in MPE was selectively elevated in LUAD with EGFR^wt^ (18.85 ± 3.53%, *n* = 28, *P* = 0.04) and EGFR-TKI acquired resistance (AR-EGFR-TKI) (19.43 ± 4.26%, *n* = 19, *P* = 0.04), compared with firstly-diagnosed EGFR^mu^ (4.24 ± 2.39%, *n* = 12). **B** Prediction of the frequency of pleural CD39 + CD8 + T cells indicating the occurrence of EGFR mutation in LUAD-related MPE. The AUC was 0.78 (*P* < 0.01) with a sensitivity and specificity of 83.33% and 71.40% when the cut-off value was 4.94%

Remarkably, as depicted in Fig. 2A, we observed a selective elevation of pleural CD39 + CD8 + T cells in LUAD with EGFR^wt^ (18.85 ± 3.53%) compared to those with initially diagnosed EGFR mutations (EGFR^mu^, 4.24 ± 2.39%, *P* = 0.04). Notably, an aberrant accumulation of pleural CD39 + CD8 + T cells was visualised in EGFR^mu^ MPE following acquired resistance to EGFR-TKI (19.43 ± 4.26%, *P* = 0.04). These findings suggest that the EGFR gene can influence the expression of CD39 by CD8 + T cells, marking a specific subset of CD8 + T cells in LUAD-related MPE.

Furthermore, we analysed the frequency of pleural CD39 + CD8 + T cells in predicting the occurrence of EGFR mutations among initially diagnosed LUAD patients. As illustrated in Fig. 2B, the ROC curve indicated a cut-off score of 4.94%. It means that when the radio of CD39 + CD8 + T cells was set at 4.94%, patients could be further classified as EGFR^mu^ or EGFR^wt^ with a sensitivity of 83.33% and a specificity of 71.40%. Accordingly, the AUC value of this ROC curve was 0.78 (95% [CI] 0.64–0.92), demonstrating that the determination of pleural CD39 + CD8 + T cells alone can yield reliable results for predicting the occurrence of EGFR mutations in LUAD.

### Pleural CD39 + CD8 + T cells exhibit an inherent phenotype associated with exhaustion

**Fig. 3:**
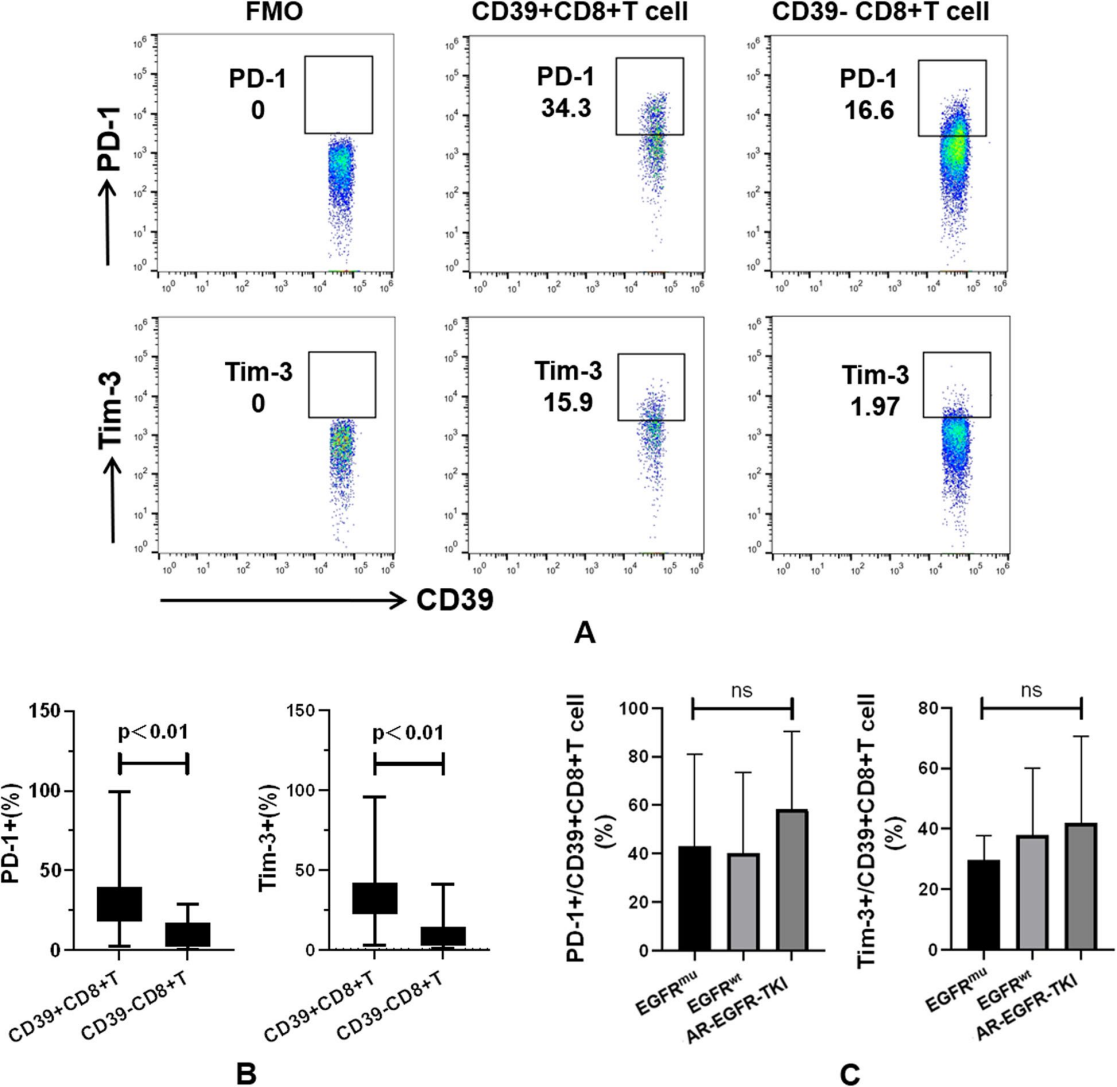
Analysis of exhaust phenotype in CD39 + CD8 + T cell. **A** Representative dot plots showing staining for PD-1 and Tim-3 on CD39 + CD8 + T cells. **B** CD39 + CD8 + T cells expressed higher levels of PD-1 (31.95 ± 4.74% vs 10.07 ± 1.62%, *P* < 0.01) and Tim-3 (35.10 ± 3.56% vs 10.28 ± 1.93%, *P* < 0.01) compared to their CD39-CD8 + T cell counterparts. **C** CD39 + CD8 + T cells from various MPE samples displayed comparable PD-1 and Tim-3 expression

The expression of PD-1 and Tim-3 has been described as a hallmark of CD8 + T cell exhaustion in cancer and chronic infections. To gain a deeper understanding of the functionality of MPE-associated CD39 + CD8 + T cells, we conducted staining for PD-1 and Tim-3 on CD39 + CD8 + T cells. As shown in Fig. 3A–B, CD39 + CD8 + T cells exhibited a higher percentage of the PD-1 + population (31.95 ± 4.74% vs 10.07 ± 1.62%, *P* < 0.01) and the Tim-3 + population (35.10 ± 3.56% vs 10.28 ± 1.93%, *P* < 0.01) compared to CD39-CD8 + T cells.

To assess the impact of the tumour environment on the phenotypic changes of CD39 + CD8 + T cells, we performed a comparative analysis of PD-1 and Tim-3 expression on pleural CD39 + CD8 + T cells obtained from LUAD with EGFR^wt^ or initially diagnosed EGFR^mu^, as well as MPE with acquired resistance to EGFR-TKI (Fig. 3C). It was observed that CD39 + CD8 + T cells from different MPE samples displayed similar PD-1 and Tim-3 expression, suggesting that these cells exhibit inherent exhaustion characteristics and are not specific to the TME.

### Unaffected effector cytokine production by pleural CD39 + CD8 + T cells

**Fig. 4:**
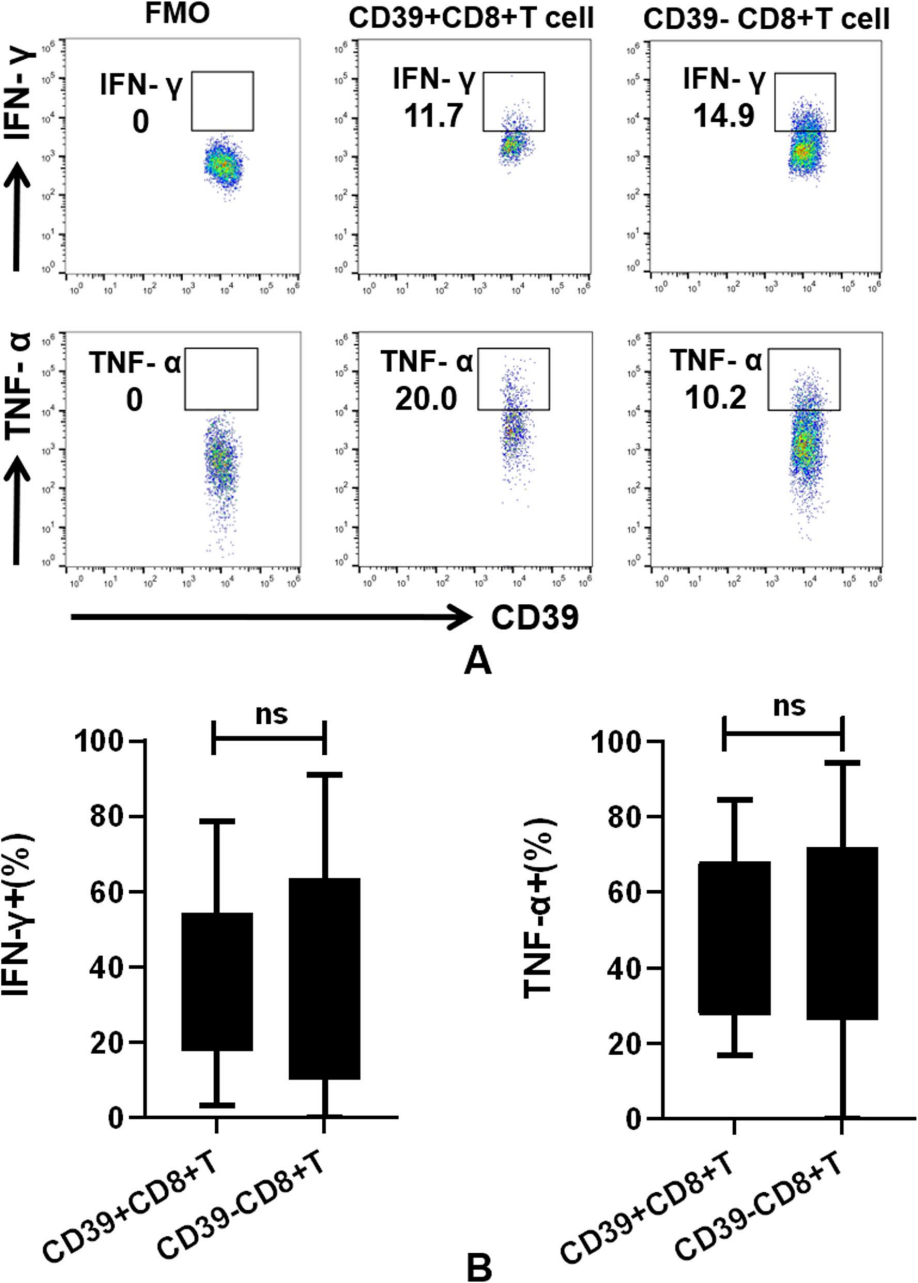
Analysis of cytolytic markers in MPE infiltrated CD39 + CD8 + T cells. **A** Representative dot plots showing staining for IFN-γ and TNF-α in CD39 + CD8 + T cells. **B** It was observed that CD39 + CD8 + T cells are capable of producing comparable levels of IFN-γ (37.02 ± 5.53% vs. 38.22 ± 7.66%, *P* = 0.75) and TNF-α (21.92 ± 6.13% vs. 23.41 ± 4.97%, *P* = 0.51) compared to CD39-CD8 + T cells

To assess whether the exhausted phenotype was associated with cytotoxicity-related cytokines, we examined the capacity of CD39 + CD8 + T cells to produce IFN-γ and TNF-α. As depicted in Fig. 4, the percentages of IFN-γ + cells (37.02 ± 5.53% vs 38.22 ± 7.66%, *P* = 0.75) and TNF-α + cells (21.92 ± 6.13% vs 23.41 ± 4.97%, *P* = 0.51) in response to phorbol 12-myristate 13-acetate-Ionomycin (PMA-IONO) stimulation were comparable between CD39 + and CD39-CD8 + T cells. This suggests that CD39 + CD8 + T cells exhibit an exhausted phenotype while retaining their cytolytic function simultaneously.

### Skewed TCR-Vβ repertoire usage in pleural CD39 + CD8 + T cell

**Fig. 5:**
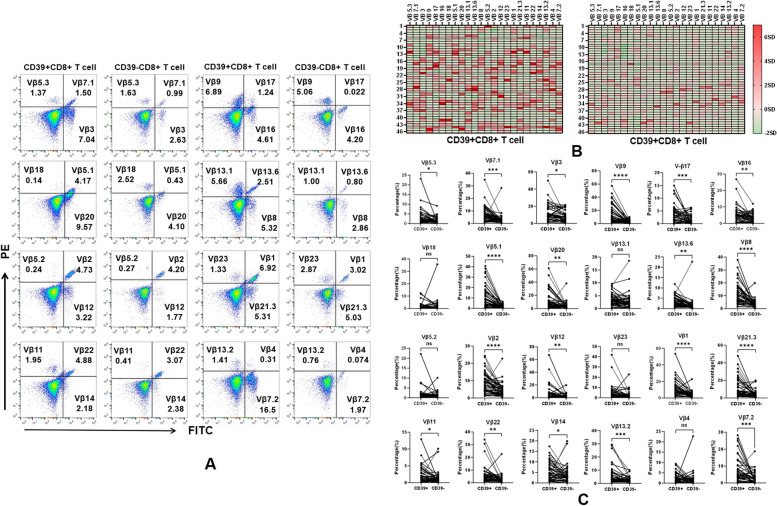
Analysis of TCR-Vβ family usage in CD39 + /CD39-CD8 + T cells. **A** Representative dot plots are shown, with each set containing three different anti-Vβ family-specific mAbs labelled with FITC, PE, or both. **B** Heatmaps displaying the expression profiles of TCR Vβ families in CD39 + and CD39-CD8 + T cells. The numbers on the left side of heatmaps represent participants, the short phrases on the top represent the usages of TCR-Vβ families, and the redder colour represents higher values of the TCR-Vβ family. **C** Individual usage of TCR-Vβ families in CD39 + and CD39-CD8 + T cells was analysed by Paired T-test

As CD39 is recognised as a useful marker for identifying tumour-reactive CD8 + T cells, we individually analysed the usage of 24 TCR-Vβ families in pleural CD39 + CD8 + T cells (Fig. 5A). The data revealed a prominent frequency of TCR-Vβ clones in pleural CD39 + CD8 + T cells compared to those observed in CD39-CD8 + T cells (Fig. 5B–C). Specifically, 19 out of the 24 TCR-Vβ families were significantly enriched in CD39 + CD8 + T cells compared to their CD39- counterparts. These findings suggest that CD39 + CD8 + T cells exhibit significantly higher TCR clonality, indicating their programming as tumour-reactive CD8 + T cells.

### EGFR-TKI acquired resistance enhances clonality of TCR-Vβ repertoire in pleural CD39 + CD8 + T cells

**Fig. 6:**
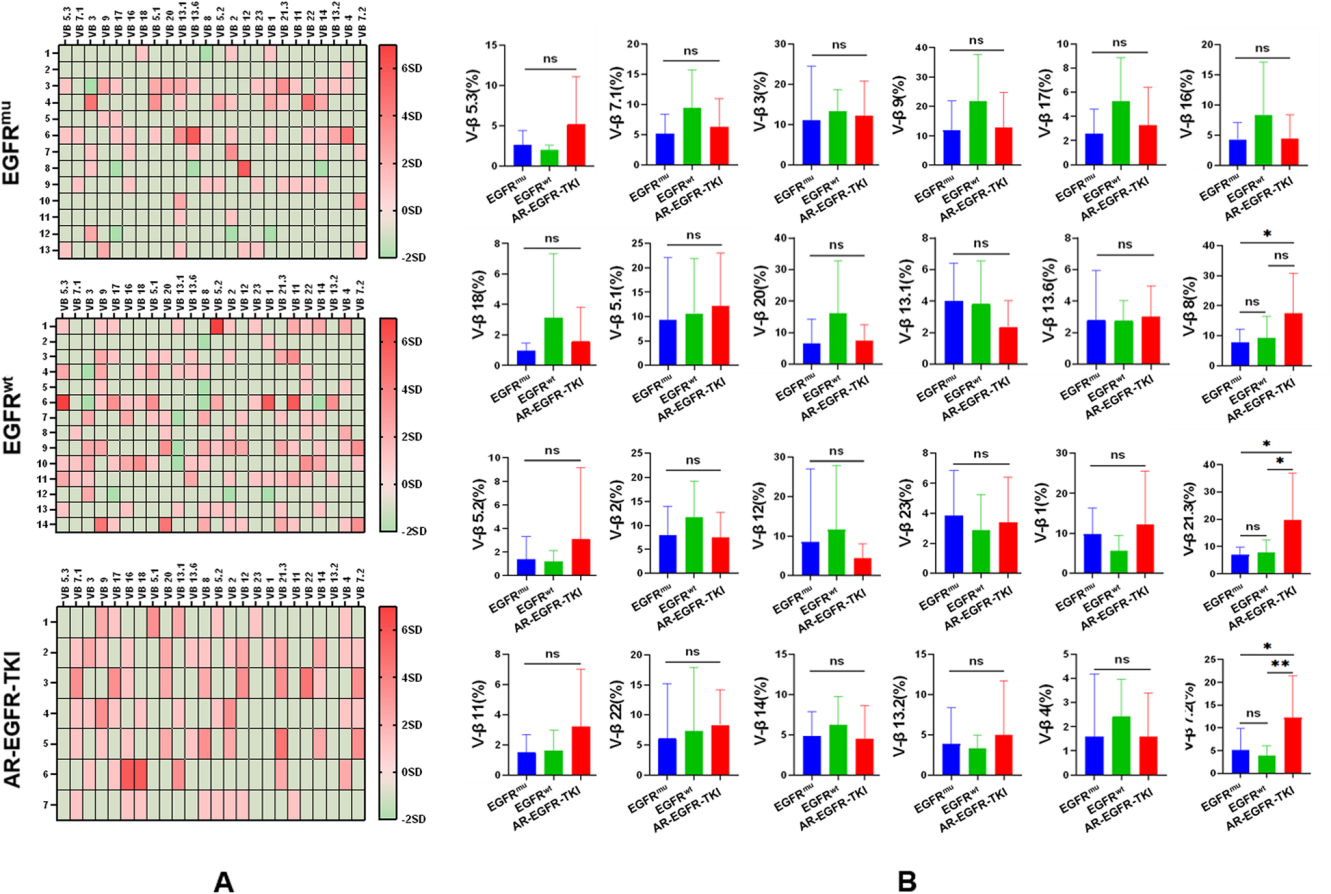
Analysis of TCR-Vβ family usage in pleural CD39 + CD8 + T cells. **A** Heatmaps displaying the expression profiles of TCR-Vβ families in pleural CD39 + CD8 + T cells. The numbers on the left side of heatmaps represent participants, the short phrases on the top represent the usages of TCR-Vβ families, and the redder colour represents higher values of the TCR-Vβ family. **B** More clones with higher frequencies can be found in pleural CD39 + CD8 + T cells from MPE with EGFR-TKI acquired resistance

Building on our earlier findings, which suggest that CD39 + CD8 + T cells may be enriched for cells recognising antigens within the tumour, we investigated whether EGFR-TKI acquired resistance could influence the TCR-Vβ diversity in pleural CD39 + CD8 + T cells. As illustrated in Fig. 6, it was observed that TCR-Vβ repertoire usage tended to be more enhanced in pleural CD39 + CD8 + T cells from patients with EGFR-TKI acquired resistance than in those with LUAD with EGFR^wt^ or initially diagnosed EGFR^mu^. This supports the hypothesis that a higher tumor mutational burden (TMB) presented by EGFR-TKI acquired resistance results in an increase of immunogenic neo-antigens, in turn affects the TCR clonality of CD39 + CD8 + T cells in MPE.

## Discussion

Considering that MPE represents the TME and involves changes in immune cells, it offers an alternative non-invasive specimen source [2]. In this context, we have identified a specific subset of tumour-infiltrating CD8 + T cells in MPE. It was observed that pleural CD39 + CD8 + T cells were selectively elevated in LUAD with EGFR^wt^ compared to initially diagnosed EGFR^mu^, and were abnormally more represented in MPE with EGFR-TKI acquired resistance. Our analysis showed that pleural CD39 + CD8 + T cells exhibit an exhausted phenotype and retained cytolytic function, along with lower TCR diversity compared to CD39-CD8 + T cells. Notably, our study revealed that the frequencies of TCR-Vβ clones tended to be more enhanced in pleural CD39 + CD8 + T cells from MPE with EGFR-TKI acquired resistance. This increased TCR-Vβ clonality upon EGFR-TKI acquired resistance may reflect altered tumour responsiveness, serving as the cellular basis for immunotherapy. The molecular mechanisms underlying the generation and regulation of CD39 + CD8 + T cells in MPE remain unknown. Previous studies have reported that CD39 expression is increased under hypoxic conditions and by TGF-β [15, 16]. Others have reported that CD39 expression is detected on CD8 + T cells with hallmarks of chronic antigenic stimulation at the tumour site [11]. In this study, we hypothesise that a low TMB presented by EGFR-mutated NSCLC results in a lack of immunogenic neoantigens, which in turn decreases the numbers of CD39 + CD8 + T cells in MPE. This hypothesis is consistent with findings in melanoma and microsatellite instability (MSI) high colon cancer, both of which possess a high mutational burden [16–18]. However, Isomoto et al. enrolled 138 EGFR-mutated patients and demonstrated that the tumour mutation burden increased after EGFR-TKI treatment, potentially leading to elevated pleural CD39 + CD8 + T cells in response [19]. In summary, these variances in CD39 expression may be explained by the genetic background of tumours, suggesting that EGFR mutations integrate immunological, metabolic, and environmental signals to regulate the immune response [20, 21].

Due to their pivotal role in the regulation of T-cell responses, PD-1 and Tim-3 are recognised as immune checkpoint molecules [22]. In this context, the majority of CD39 + CD8 + T cells in malignant pleural effusion (MPE) exhibited the exhausted phenotype typical of T cells, as indicated by the expression of PD-1 and Tim-3. However, we also observed that these cells demonstrated unimpaired production of IFN-*γ* and TNF-*α*, which are characteristic features of a Tc1-like phenotype. This observation suggests that CD39 + CD8 + T cells differ from other well-known exhausted T cell subsets and argues for CD39 as an activation marker rather than an exhaustion marker. As further support, a recent study has reported that T cell dysfunction is epigenetically imprinted and that two dysfunctional states exist: a “plastic” state from which T cells can be rescued and a “fixed” state in which T cells are resistant to reprogramming [23, 24]. Therefore, when considering immunotherapeutic strategies, this provides a new understanding of how to address the dysfunction or exhaustion of T cells in MPE. It also implies that targeting CD39 + CD8 + T cells represents a promising therapeutic approach to enhance immune responses against tumours [25, 26].

Identifying tumour antigen-specific T cells from cancer patients holds significant implications for immunotherapy. In our present study, we employed TCR-Vβ repertoire analysis to investigate selective T-cell responses against tumour antigens. Our findings align with the observations made by Ting Liu and colleagues, who described how high-affinity neoantigens can trigger anti-tumour activity by activating tumour-reactive CD39 + CD8 + T cells [27]. Thomas Duhen also demonstrated that CD103 + CD39 + CD8 + TILs possess a distinct TCR repertoire and can be identified as tumour-reactive CD8 + T cells in human solid tumours [28]. Additionally, Simoni and colleagues found that CD8 + T cells specific to tumour neoantigens exhibit high CD39 expression [29]. In a phase III clinical trial for non-small cell lung cancer (NSCLC), a gene signature of CD39 + CD8 + T cells was shown to predict the benefit from immune checkpoint blockade (ICB) but not chemotherapy. Since improved identification of anti-tumour T cells is crucial for advancing cancer immunotherapies, the anticipated efficacy of pleural CD39 + CD8 + T cell-based immunotherapy could be attributed to both their cytolytic activity and their tumour-reactive characteristics [30].

Despite the significant antitumor efficacy of immunotherapy in advanced NSCLC, the results in patients harbouring activating EGFR mutations have been disappointing. The biological mechanisms underlying both unresponsiveness and resistance to immunotherapy in EGFR^mu^ NSCLC patients have been partially investigated. EGFR-mutated NSCLC lacks immunogenic neoantigens, leading to a “lymphocyte depletion” phenotype in the TME. This has led to the hypothesis that the generation and evolution of the TCR repertoire are impacted in this T cell population. In this context, we have highlighted CD39 + CD8 + T cells as a proxy for tumour-reactive CD8 + T cells in human lung cancer, emphasising their key role as modulators of immunotherapy. We have also found that the number of pleural CD39 + CD8 + T cells is associated with acquired resistance to TKI in adenocarcinoma. Theoretically, targeted therapy may lead to the release of neoantigens through tumour cell death, resulting in immunomodulation that can potentiate immune responses [31]. The increase in CD39 + CD8 + T cells in the TME is suggested as a potential mechanism involved in immunotherapy for EGFR^mu^ NSCLC patients with third-generation EGFR-TKI acquired resistance. Shun Lu et al. enrolled 444 patients and found that immunotherapies plus bevacizumab and chemotherapy was associated with a statistically significant and clinically meaningful improvement in patients with EGFR-mutated non-squamous non-small-cell lung cancer who progressed on EGFR tyrosine-kinase inhibitor therapy [32]. We [18] believed that EGFR-TKI treatment was associated with changes in the TME of LUAD with EGFR^mu^, and change of clonality of CD39 + CD8 + T cell would provide clues for optimization of subsequent immunotherapy.

In summary, our findings indicate that EGFR mutations in MPE lead to an altered immune regulatory phenotype in CD8 + T cells, which is associated with CD39 expression. This offers a novel insight into immune regulation within MPE and has implications for defining CD8 + T cell subsets as potential biomarkers and targets for immunotherapeutic interventions. Understanding the defined state of CD8 + T cells may hold the key to elucidating the relationship between targeted therapy and immunotherapy in MPE. Furthermore, among the numerous predictive biomarkers explored for immunotherapy, it would be of interest to investigate changes in the frequency and/or activation status of CD39 + CD8 + TILs in lung cancer patients responding to anti-PD-1 therapies.

## Data Availability

The datasets used and analysed during the current study are available from the corresponding author upon reasonable request.
